# A Novel Photosensitizer 3^1^,13^1^-phenylhydrazine -Mppa (BPHM) and Its *in Vitro* Photodynamic Therapy against HeLa Cells

**DOI:** 10.3390/molecules21050558

**Published:** 2016-04-29

**Authors:** Wenting Li, Guanghui Tan, Jianjun Cheng, Lishuang Zhao, Zhiqiang Wang, Yingxue Jin

**Affiliations:** Key Laboratory for Photonic and Electronic Bandgap Materials, Ministry of Education, College of Chemistry & Chemical Engineering, Harbin Normal University, Harbin 150025, China; 13039864430@163.com (W.L.); 13504800928@163.com (J.C.); hsdzls@163.com (L.Z.)

**Keywords:** photodynamic therapy (PDT), BPHM, cell cytotoxicity

## Abstract

Photodynamic therapy (PDT) has attracted widespread attention due to its potential in the treatment of various cancers. Porphyrinic pyropheophorbide-a (PPa) has been shown to be a potent photosensitizer in PDT experiments. In this paper, a C-3^1^,13^1^ bisphenylhydrazone modified methyl pyropheophorbide-a (BPHM) was designed and synthesized with the consideration that phenylhydrazone structure may extend absorption wavelength of methyl pyro-pheophorbide-a (Mppa), and make the photosensitizer potential in deep tumor treatment. The synthesis, spectral properties and *in vitro* photodynamic therapy (PDT) against human HeLa cervical cancer cell line was studied. Methyl thiazolyl tetrazolium (MTT) assay showed the title compound could achieve strong inhibition of cervical cancer cell viability under visible light (675 nm, 25 J/cm^2^). Cell uptake experiments were performed on HeLa cells. Morphological changes were examined and analyzed by fluorescent inverted microscope. In addition, the mechanism of the photochemical processes of PDT was investigated, which showed that the formation of singlet oxygen after treatment with PDT played a moderate important role.

## 1. Introduction

Cancer has been seriously threatening human health for a long period. Diagnosis and treatment of cancer has become the most pressing concern in contemporary society. Many therapeutic methods have been developed over the past decades, such as chemotherapy, radiotherapy and surgical therapy. Each of these approaches has disadvantages and drawbacks, such as incomplete treatment, side effects, patient suffering and high cost, and one should balance the disadvantages and the therapeutic effect. Among various choices for cancer treatment, photodynamic therapy (PDT) as a noninvasive therapeutic modality providing painless and repeating treatment for patients, has recently obtained regulatory approval for various cancers, including skin tumors [1], non-small-cell lung cancer [2], urinary system tumors [3], breast cancer [4], head and neck cancer [5], digestive system tumors [6]; hence, PDT has a good foreground and will provide alternative therapeutic methods.

In PDT, the photosensitizer would attack any tissues it encounters. Because the photosensitizer may be excited by appropriate wavelength of light, it then passes on the excess energy to surrounding molecular oxygen, resulting in the generation of reactive oxygen species (ROS) [7]. These ROS are free radicals (Type I PDT) that generated through electron transfer from a substrate molecule, or highly reactive state of oxygen known as singlet oxygen (Type II PDT). Photosensitizer is a critical factor in PDT. An ideal photosensitizer should not only have high efficiency in generating ROS within the aerobic tissues but also have characteristics of low dark toxicity, easily targeted aggregation and long wavelength absorption (>600 nm) [8]. The earliest photosensitizers applied in clinic are hematoporphyrin derivative (HPD) and ‘photofrin’ [9,10], which has a number of shortcomings, such as complicated components, weak absorption band in the red region (630 nm), long-term cutaneous photosensitivity (about three months) and prolonged *in vivo* metabolism. Subsequently developed hematoporphyrin monomethyl ether (HMME) and 5-Aminolevulinic acid (5-ALA) in the 1990s were single component photosensitizers with better stability than HPD and ‘photofrin’ [11,12], yet they were not suitable for deep-seated tumors for the light being absorbed by the photosensitizers (<630 nm) could not penetrate deep tissue. With the aim of overcoming some of the disadvantages, considerable effort has been put into the development of new photosensitizers. Some derivatives of porphyrins [13,14], phthalocyanines [15,16] and chlorin [17,18] had been used as photosensitizers. These photosensitizers showed better biocompatibility, bioavailability, target-ability and faster metabolism [19,20,21,22]. 

Methyl pheophorbide-a (Mpa) and methyl pyro-pheophorbide-a (Mppa) as chlorin analogues are ideal materials for photodynamic therapy due to their advantages of long absorption wavelength (>667 nm), low dark toxicity, high molar extinction coefficient and high rate of fluorescence quantum yield [23]. C-3, 5, 7, 10, 12, 13, 17, 20 of MPPa are high-active reactive sites and are easily modified for the development of new photosensitizers. On the other hand, it is widely acknowledged that increasing the π-conjugation extending from the porphyrin core can lead to enhanced absorption properties [24]. In this present study, we made a modification on C-3, C-13 of Mppa by *p*-CF_3_-phenylhydrazine to produce a new chlorin-based compound to develop photosensitizers in PDT. We consider that modification by *p*-CF_3_-phenylhydrazin may increase the π-conjugation extending from the porphyrin core and shift absorption towards longer wavelengths. In general, the longer the wavelength, the deeper the penetration into tissues [25,26]. Red light can be used successfully for deeper localized targets; consequently, compounds with longer absorption wavelengths can be used in the treatment for deeper tissues.

In this paper, the synthesis, the UV-visible spectroscopy and fluorescence spectroscopy of C-3^1^,13^1^ bisphenylhydrazone modified methyl pyropheophorbide-a (BPHM) were studied. Moreover, *in vitro* photodynamic therapy (PDT) against the human HeLa cervical cancer cell line was studied by MTT assay to evaluate the title compound as the photosensitizer agent in support of PDT. Cell uptake experiments were performed in order to investigate the intracellular distribution. Morphological changes of HeLa cells after PDF treatment were analyzed by fluorescent inverted microscope. In addition, the photochemical processes mechanism of PDT was investigated by using specific quenching agent sodium azide (SA) and D-mannitol (DM) [27,28], respectively.

## 2. Results and Discussion

### 2.1. Chemistry

**Scheme 1:**
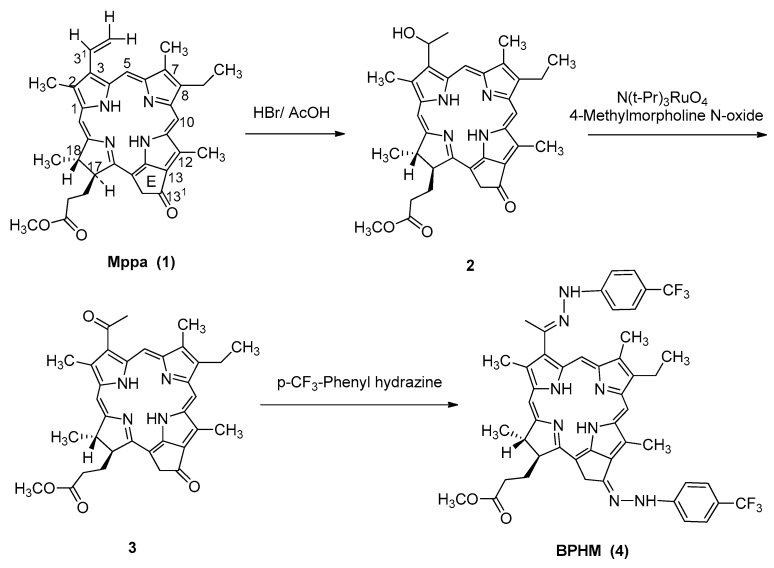
The synthesis of BPHM (C-3^1^,13^1^-Bisphenylhydrazone).

The starting material Methyl pyropheophorbide-a (Mppa) was synthesized according to our previously reported procedure [29] (Scheme 1). Firstly, Mppa was hydrolyzed by hydrobromic acid in acetic acid, then oxidized by N(*t*-Pr)_3_RuO_4_ to obtain C-3^1^,13^1^-dicarbonyl product (**3**), which reacted with *p*-CF_3_-phenylhydrazine under reflux conditions to obtain the end-product C-3^1^,13^1^-Bisphenylhydrazone modified methyl pyropheophorbide-a (BPHM, **4**). In practice, we initially performed the reaction under a moderately acidic condition at room temperature, yet it took a long time (>24 h) to complete the reaction. We assumed the conjugation between the carbonyl groups and cyclic π-aromatic system in the compound reduced the activity of nucleophilic addition. When compound **3** reacted with *p*-CF_3_-phenylhydrazine under reflux, it only needed 5 h to obtain the target compound with a high yield.

### 2.2. Optical Properties

Appropriate wavelength of light penetrates healthy tissue to reach the lesion, then activates the photosensitizer in focal tissues, leading to damage. Modifications by phenylhydrazone on C-3^1^,13^1^ of Mppa lead to compounds with longer absorption wavelength, for it can effectively increase the π-conjugation system extending from the porphyrin core, resulting in enhanced absorption properties. Hence, it is necessary to investigate the optical properties of BPHM.

**Figure 1:**
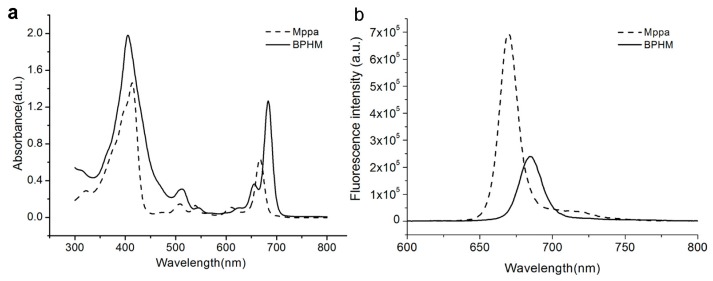
Absorption (**a**) and fluorescence spectra (**b**) of Mppa, BPHM in CH_3_OH (2.15 × 10^−5^ M for UV absorption spectrum, 2.5 × 10^−7^ M for fluorescence spectrum).

Figure 1a shows the ultraviolet-visible absorption of Mppa and BPHM recorded in MeOH (2.15 × 10^−5^ M), respectively. Both Mppa and BPHM demonstrate the characteristic adsorption of chlorins, which absorb throughout the ultraviolet region into the visible region between about 300 and 800 nm. Strong Soret absorption peaks appear in the range 350–420 nm. The Soret absorption peak of BPHM increased obviously compared with Mppa, yet the absorption wavelength is not changed. For BPHM, four Q peaks appear in visible area. The strongest absorption is Qy peak, which displays a marked 16 nm redshift and an obvious increase of peak intensity compared to Mppa. The molar extinction coefficient is 5.88 × 10^4^ L/mol·cm calculated based on Beer-Lambert Law.

Because of the four conjugated pyrrole rings, porphyrins possess high rigidness and better coplanarity, exhibiting strong fluorescence at room temperature. After being excited by light, the emitted visible light from photosensitizers is very helpful in photodynamic clinical diagnosis. Figure 1b shows the fluorescence spectrograms of Mppa and BPHM recorded in MeOH (2.5 × 10^−7^ M), respectively. The obtained fluorescence spectra of Mppa and BPHM are both symmetric after being excited by 413 nm visible light, and the emission wavelength are 670 nm and 685 nm, respectively.

### 2.3. Photodynamic Activities

After a photosensitizer is promoted to an excited state under light, it will produce singlet oxygen, which would lead to high cytotoxicity to target cells. In this part, HeLa cells were used to detect the anticancer activity of our designed compound. The viability of HeLa cells incubated with different concentrations of BPHM and Mppa were analyzed by MTT assay. 

**Figure 2:**
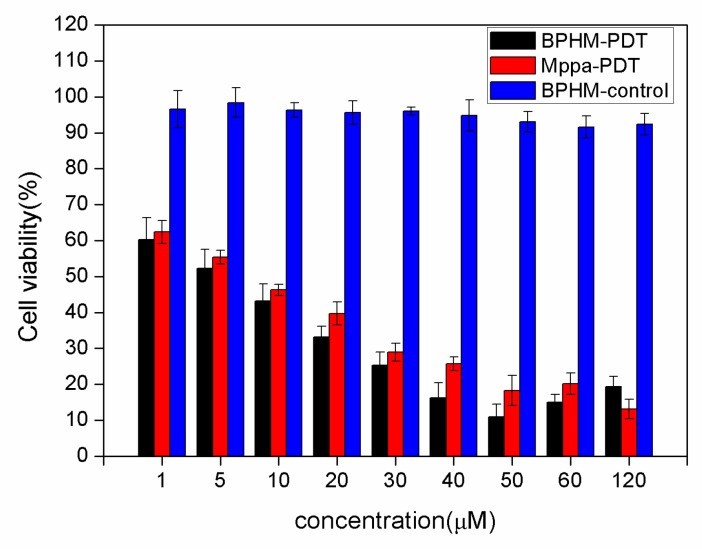
Cell viability of three groups: BPHM experiment groups (black columns), Mppa experiment groups (red columns) and control groups (blue columns). Each group was cultured with different concentrations of BPHM or Mppa (1, 5, 10, 20, 30, 40, 50, 60 and 120 μM, 200 μL), cell viability was assessed by MTT assay after 24 h. Statistically significant between BPHM and Mppa experiment groups were performed by *t*-test, *p* < 0.05 showed the difference was significant).

Figure 2 showed the photodynamic activity of the title compound and Mppa against HeLa cells. Black columns stand for the cell viability of BPHM experiment groups, red columns stand for the cell viability of Mppa experiment groups, and blue columns for the cell viability of control groups. The control groups (blue columns) treated with different concentrations of BPHM (1, 5, 10, 20, 30, 40, 50, 60 and 120 μM) without light show high cell viability (>90%), which suggests that BPHM could hardly influence the cancer cell without light. However, the BPHM and Mppa experiment clearly showed that with the increasing concentrations of BPHM and Mppa, the cell viability rate is on the decline, suggesting that both BPHM and Mppa have good cell growth inhibiting ability. When the concentration of BPHM reached to 50μM, the cell viability lowed to 10.99% ± 1.26%, suggesting that the BPHM had strong photodynamic effects on the HeLa cells. Moreover, the cell viability of Mppa was slightly higher than BPHM (*p* < 0.05, the difference was significant), and the IC_50_ values of BPHM and Mppa under visible light (675 nm, 25 J/cm^2^) are 9.21 ± 0.91 μM and 12.90 ± 0.53 μM, respectively. Although our designed compound did not show an obvious advantage compared to Mppa, it has longer absorption wavelength than that of Mppa, which gives it more potential in deep tumor treatment. In addition, due to the phenylhydrazine part, our compound possesses relatively high lipid solubility, which makes it easier to permeate cell membranes and enter the cells. All in all, our designed compound has long absorption wavelength and slightly higher cell toxicity than Mppa. BPHM could kill the cell effective under the light and the low dark toxicity provides the feasibility in clinical application.

### 2.4. Formation of Reactive Oxygen Species in PDT 

**Figure 3:**
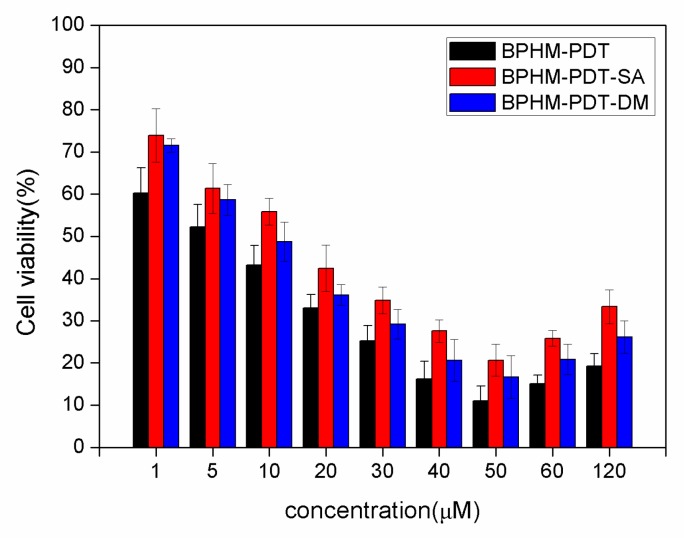
Cell viability of BPHM-PDT, BPHM-PDT-SA, and BPHM-PDT-DM group after PDT treatment 24 h, respectively. BPHM-PDT (black column) stands for the groups treated with BPHM under light (675 nm). BPHM-PDT-SA (red column) stands for the groups treated with BPHM and Sodium azide under light (675 nm). BPHM-PDT-DM (blue column) stands for the groups treated with BPHM and d-mannitol under light (675 nm). All of the groups were cultured with different concentrations of BPHM (1, 5, 10, 20, 30, 40, 50, 60 and 120 μM, 200 μL), the concentration of SA was 20 mmol/L, DM was 40 mmol/L Cell viability was determined by methyl thiazolyl tetrazolium (MTT) assay. A *t*-test was performed according to the IC_50_ of each group. *p* < 0.05 (*t*-test) between BPHM and BPHM-PDT-SA experiment groups showed the difference was significant; *p* < 0.05 (*t*-test) between BPHM and BPHM-PDT-DM experiment groups showed the difference was significant; *p* < 0.05 (*t*-test) between BPHM-PDT-SA and BPHM-PDT-DM experiment groups showed the difference was significant.

In order to investigate the mechanism of photochemical processes (Type I and Type II) in PDT, corresponding ROS of Type I and Type II generated from photodynamic reaction were quenched by using specific quenching agent sodium azide (SA) and d-mannitol (DM), respectively [27,28]. Sodium azide (SA) and D-mannitol (DM) could selectively react with oxygen free radicals and singlet oxygen (^1^O_2_), respectively, making the corresponding ROS inoperative on cancer cells. Figure 3 showed the influences of three different PDT processing methods on cytotoxicity effects. The cell viability of BPHM-PDT-SA group and BPHM-PDT-DM group was a little higher than that of the BPHM-PDT group, suggesting that the key ROS have been quenched, resulting in low cytotoxicity to HeLa cells. Moreover, the cell viability of BPHM-PDT-SA group was slightly higher than that of BPHM-PDT-DM group at various concentrations (1–120 μM) of BPHM, demonstrating that *in vitro* Type I and Type II photodynamic reactions occurred simultaneously, yet Type II reaction played a moderate important role.

As mentioned earlier, in the Type I photodynamic reaction, photosensitizer could interact with any active substrate, producing hydroxyl radicals (HR), superoxide anion, or hydrogen peroxide by electron transfer process. We inferred that the cancer cells had provided some integral substrate for photosensitizer to complete the Type I reaction during the in vitro test. For Type II reaction, the excitation energy might be transferred to nearby tissue oxygen, producing singlet oxygen, that is, the Type II reaction would take place generally in the presence of tissue oxygen, yet in our cell experiments, the excitation energy might be transferred to cellular components. Therefore, coexistence of Type I and Type II was reasonable in practice.

### 2.5. Cellular Uptake

In order to be used as a potential anticancer agent, a photosensitizer should firstly be able to enter cells. Successful application of photosensitizer for PDT needs the effective uptake of them by cells. Hence, studying the interaction of a photosensitizer with a cell membrane for effective cellular uptaking seems to be important. Cellular uptake is usually a prerequisite and is influenced by size, shape, material, surface charge, and surface hydrophobicity. Generally, highly lipid soluble drugs can penetrate the plasma membrane easily. Due to the phenylhydrazine structure in our designed compound, it may possess better fat-solubility; hence, it may enter the cells easily. Generally, in the uptaking test, the nucleus was firstly stained by 4′,6-diamidino-2-phenylindole (DAPI, a blue-fluorescent DNA stain) solution, after which the nucleus can be easily observed due to its bule fluorescence. If the substance being examined could be uptaken successfully by HeLa cells, the cells may display the corresponding color of fluorescent substances obviously. 

**Figure 4:**
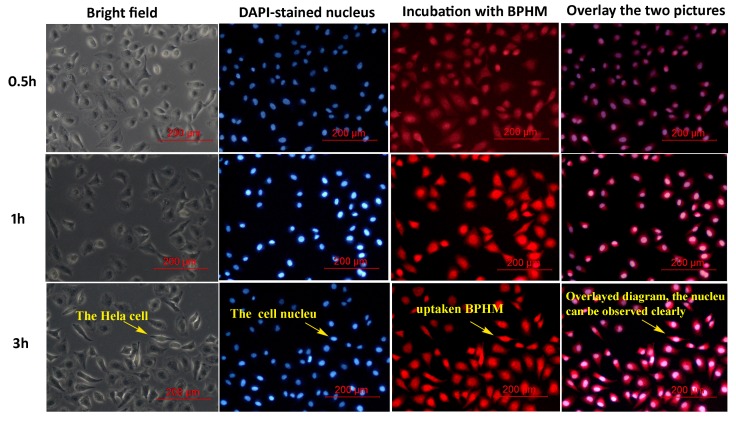
Fluorescence inverted microscopic images of cellular uptake test: HeLa cells were incubated with BPHM (1 mL, 50 μg/mL) at 37 °C for 30 min, 1 h, and 3 h, respectively. The nucleus was stained with DAPI (**blue**), the red fluorescence was due to BPHM. The corresponding cells states were observed in bright field.

In the present study, cell uptaking tests were analyzed by fluorescence imaging. Our compound emits red fluorescence under fluorescent inverted microscope due to its large conjugated system. As shown in Figure 4, the nucleus of a HeLa cell stained by DAPI dye solution showed blue fluorescence, and the red fluorescence was due to BPHM signal. After being incubated with BPHM for 30 min, the red fluorescence was observed from many of the cells, suggesting that some BPHM began to enter the cells. After being incubated for 1 h, most of the cells showed red fluorescence, and the signal intensity was stronger than that observed at 30 min. After being incubated for 3 h, the red fluorescent signal can be detected from almost all cells, and the signal intensity was stronger than before. From what has been discussed above, we inferred that BPHM can be successfully uptaken by HeLa cells. It is noteworthy that BPHM could quickly enter the cells in less than 0.5 h, suggesting that the photosensitizer could take effect more quickly in practical application, which also provides the feasibility in clinical application. We inferred that the approprite lipid-water partition coefficient of BPHM plays an important role in entering cells. In addition, the cells showed marked red fluorescence after uptake BPHM in cancer cells, which makes the compound a potential agent for medical fluorescence imaging.

Considering that BPHM can rapidly enter cells, and possessed the hydrophobic character due to the phenylhydrazine structure, hence it may agglomerate when subjected to aqueous solution and may enters cells at the nano-scale [30,31,32]. In order to investigate how BPHM enters cells (in molecular states or nanoparticles), we performed dynamic light scattering (DLS) measurement in phosphate buffer saline. DLS is an important tool for characterizing the size of nanoparticles, and measuring the agglomeration state of nanoparticles as a function of time or suspending solution. The results showed that BPHM have a diameter of around 150 nm, suggesting that they enter cells at the nano-scale (Figure S1, shown in Supplementary).

### 2.6. Morphological Changes of HeLa Cells after PDT 

**Figure 5:**
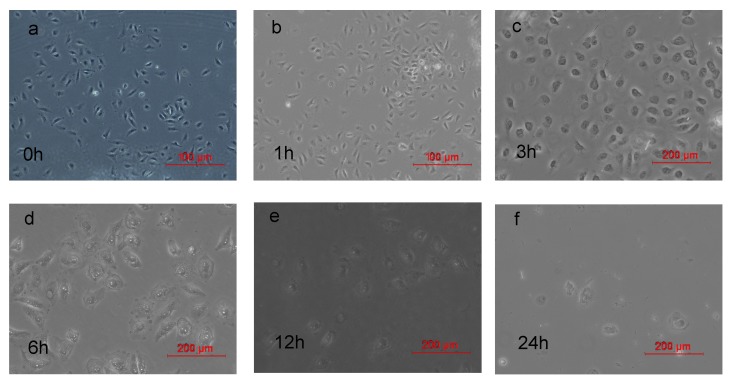
Morphological changes of HeLa cells incubated with 30 μM BPHM after PDT: (**a**) 0 h; (**b**) 1 h; (**c**) 3 h; (**d**) 6 h; (**e**) 12 h; (**f**) 24 h.

The cell phenotype was analyzed by fluorescent inverted microscope (FIM) after irradiation with light for 1, 3, 6, 12, 24 h, respectively, compared with the cell without any treatment. As shown in Figure 5a (0 h), HeLa cells without any treatment are generally spindle-shaped with high refractive index and steady cytoplasm, and almost all grew well attached on the plate. However, after being treated with PDT and cultured for an additional 3 h (Figure 5c), the cells gradually become rounder, with shading degree decreased. After 6 h (Figure 5d), injury of the membrane structure and dilation of intercellular space were observed, with decreased cell adhesive ability. During prolonged culture (Figure 5e,f), almost all the cells died. Morphological variation of cells clearly indicated that treatment with BPHM under light could result in damage or cell death in HeLa cells.

## 3. Experimental Section

### 3.1. General Remarks

All chemicals were used as received without further purification. All solutions were freshly prepared with deionized water. Sodium azide (SA) and d-Mannitol (DM) were bought from Sinopharm Chemical Reagent Co., Ltd. (Shanghai, China). Chlorophyll paste was bought from Shandong Guangtongbao Pharmaceuticals Co., Ltd. (Qingzhou city, Shandong, China). Dulbecco’s modified eagle medium (DMEM), penicillin, fetal bovine serum (FBS), and streptomycin were purchased from Beijing Dingguo Biotechnology Co. (Beijing, China). Dimethyl sulfoxide (DMSO) and 3-(4,5-dimethylthiazol-2-yl)-2,5-dipheny-ltetrazolium bromide (MTT) were purchased from Sigma (Shanghai, China). Phosphate buffered saline (PBS) purchased from Invitrogen (Beijing, China) was used as a balanced salt solution in cell culture. PBS used in other experiments was prepared by mixing stock solutions of NaH_2_PO_4_ and Na_2_HPO_4_. Particle size measurements were performed in phosphate-buffered saline on a ZetaPALS analyzer at 25 °C, and the sample concentration in PBS was 2 × 10^−7^ M. The measurements were made at a detection angle of 90°.

All the chemical reactions were performed under nitrogen atmosphere and away from sunshine. ^1^H-NMR and ^13^C-NMR spectra were recorded at 400 and 100 MHz, respectively, on an AMX400 spectrometer (Bruker, Bremen, Germany) with tetramethylsilane (TMS) as an internal standard. Mass spectra were recorded with a Hitachi VG-7070 spectrometer (Hitachi, Manchester, UK). UV-Vis absorption and emission spectra were recorded using LAMBDA 25 spectrometer (PerkinElmer, Shanghai, China) and spectrofluorophotometer with a 150 W xenon lamp as a visible excitation light source (RF-5301PC, Shimadzu, Columbia, MD, USA), respectively. All spectra were obtained in a quartz cuvette (path length = 1 cm). The excitation and emission slit widths were both 10 nm, and photomultiplier tube (PMT) voltage of 700 V. The UV-Vis absorption spectrum of BPHM was recorded in methanol (2.15 × 10^-5^ M) in the 300–800 nm range. The fluorescence intensities/spectra were measured at λ ex/em = 412/685 nm

### 3.2. Synthesis of 3^1^-hydroxyethylMppa (***2***)

Mppa (50 mg, 0.09 mmol) was dissolved in 15 mL of 30% hydrobromic acid-ethanol solution, and stirred for 4 h at 50 °C under nitrogen atmosphere. After the reaction mixture was cooled, pour them into 100 ml water, and extract with 50 mL dichloromethane for three times. The organic layer was successively washed by 30 mL 10% Na_2_CO_3_ solution and 30 mL water, then dried by anhydrous Na_2_SO_4_, concentrated by reducing pressure to remove solvent. The residue was further purified by gel column chromatography with ethyl acetate/petroleum ether (1:1) to give a dark green solid **2** (60%).

*3^1^-hydroxyethylMppa* (**2**). UV-Vis(CH_3_OH) (relative intensity) λ_max_: 408 (1.045), 504 (0.098), 535 (0.039), 604 (0.082), 661 (0.485); ^1^H-NMR(CDCl_3_) δ: −1.91 (s, 1H, NH), −0.12 (s, 1H, NH), 1.67 (t, *J* = 7.6 Hz, 8-CH_3_), 1.76 (d, *J* = 8.4 Hz, 3H, 18-CH_3_), 2.11 (d, *J* = 6.4 Hz, 3H, 3^2^-CH_3_), 2.21–2.26 (m, 2H, 17^1^-CH_2_), 2.60–2.71 (m, 2H, 17^2^-CH_2_), 3.21 (s, 3H, 7-CH_3_), 3.37 (s, 3H, 2-CH_3_), 3.38 (s, 3H, 12-CH_3_), 3.58 (s, 3H, 17-OCH_3_), 3.61–3.68 (m, 8-CH_2_), 4.18–4.23 (m, 1H, 17-CH), 4.23–4.44 (m, 1H, 18-CH), 5.06 (d, *J* = 17.2 Hz, 1H, 13^2^-CH_2_), 5.26 (d, *J* = 19.6 Hz, 1H, 13^2^-CH_2_), 6.31–6.39 (m, 1H, 3^1^-CH), 8.49 (s, 1H, Ar-H), 9.34 (s, 1H, Ar-H), 9.61 (s, 1H, Ar-H).

### 3.3. Synthesis of 3-ethoxy-Mppa (***3***)

Compound **2** (113 mg, 0.2 mmol) was dissolved in 20 mL dried dichloromethane, and 4-Methylmorpholine N-oxide (20 mg) was then added. The mixture was stirred for 15 min, then add tetrapropylammonium perrhuthenateacetic in batches (15 mL). After stirring for 2 h, add 20 mL water. The organic layer was dried by anhydrous Na_2_SO_4_ and then concentrated by reducing pressure to remove solvent. The residue was further purified by gel column chromatography with ethyl acetate/petroleum ether (1:1) to give a black solid **3** (80%).

*3-ethoxy-Mppa* (**3**). UV-Vis(CH_3_OH) (relative intensity) λ_max_ 413 (0.158), 513 (0.199), 546 (0.180), 622 (0.122), 682 (0.858); ^1^H-NMR (CDCl_3_) δ: −2.08 (s, 1H, NH), −0.11 (s, 1H, NH), 1.68 (t, *J* = 7.8 Hz, 8-CH_3_), 1.78 (d, *J* = 7.2 Hz, 3H, 18-CH_3_), 2.21–2.26 (m, 2H, 17^1^-CH_2_), 2.59–2.77 (m, 2H, 17^2^-CH_2_), 3.20 (s, 3H, 3^1^-CH_3_), 3.26 (s, 3H, 1-CH_3_), 3.60–3.68 (m, 11H, 7-CH_3_, 12-CH_3_, 17-OCH_3_, 8-CH_2_), 4.34–4.36 (m, 1H, 17-CH), 4.53–4.57 (m, 1H, 18-CH), 5.08 (d, *J* = 19.6 Hz, 1H, 13^2^-CH_2_), 5.26 (d, *J* = 19.6 Hz, 1H, 13^2^-CH_2_), 8.76 (s, 1H, Ar-H), 9.48 (s, 1H, Ar-H), 9.93 (s, 1H, Ar-H); ^13^C-NMR(CDCl_3_) δ: 11.2, 12.0, 13.4, 17.3, 19.37, 23.3, 29.3, 30.9, 33.4, 48.1, 49.2, 51.6, 52.1, 94.1, 100.2, 103.5, 106.7, 129.5, 131.3, 134.1, 135.3, 137.3, 138.9, 139.1, 144.8, 148.7, 151.8, 154.9, 161.3, 170.1, 173.3, 195.9, 199.2, 206.7 ; FAB-MS: 565 (M^+^ + 1); Anal calcd for C_34_H_36_N_4_O_4_: C, 72.32; H, 6.43; N, 9.92; found C, 72.64; H, 6.21; N, 10.11.

### 3.4. Synthesis of 3^1^,13^1^-bisphenylhydrazone-Mppa (**BPHM**, ***4***)

Compound **3** (62 mg, 0.11 mmol) and acetic acid (5 mL) were dissolved in 70 mL of ethanol, and *p*-CF_3_-phenylhydrazine (0.22 mmol) was added gradually with stirring under nitrogen. The solution was stirred at reflux for 7 h. Then, it was cooled to room temperature, and concentrated by reducing pressure. The raw product was purified by gel column chromatography with ethyl acetate/petroleum ether (1:1) and further recrystallized from acetate/petroleum ether (1:7) to obtain pure BPHM (85%).

*3^1^,13^1^-phenylhydrazone-Mppa* (**4**). UV-Vis(CH_3_OH) (relative intensity) λmax: 409 (0.82), 511 (0.14), 540 (0.05), 623 (0.05), 683 (0.54); ^1^H-NMR(CDCl_3_) δ: −2.87 (s, 1H, NH), −1.03 (s, 1H, NH), 1.67 (t, *J* = 7.6 Hz, 8-CH_3_), 1.83 (d, *J* = 7.2 Hz, 3H, 18-CH_3_), 2.24–2.31 (m, 2H, 17^1^-CH_2_), 2.51–2.67 (m, 2H, 17^2^-CH_2_), 3.04 (s, 3H, 3^1^-CH_3_), 3.24 (s, 3H, 7-CH_3_), 3.39 (s, 3H, 2-CH_3_), 3.60 (s, 3H, 12-CH_3_), 3.73 (s, 3H, 17-OCH_3_), 3.77–3.80 (m, 2H, 8-CH_2_), 4.50–4.54 (m, 1H, 17-CH), 4.73–4.78 (m, 1H, 18-CH), 5.47–5.63 (dd, *J*_1_ = 20.0 Hz, *J*_2_ = 19.6 Hz, 2H, 13^2^-CH_2_), 7.62–7.74 (m, 8H, Ph-H), 9.34 (s, 1H, Ar-H), 9.12 (s, 1H, Ar-H), 9.80 (s, 1H, Ar-H), 10.08 (s, 1H, Ar-H), 10.41 (s, 1H, =N-NH), 10.52 (s, 1H, =N-NH); ^13^C-NMR (CDCl_3_) δ: 11.1, 11.9, 12.4, 17.7, 18,5, 18.8, 19.5, 23.5, 28.6, 30.3, 48.3, 51.2, 52.3, 56.0, 93.5, 100.0, 100.3, 106.1, 112.4, 112.8, 118.2, 118.9, 119.1, 123.8, 123.9, 126.5, 129.8, 131.7, 135.7, 136.4, 136.8, 137.5, 140.4, 141.3, 143.3, 144.4, 145.7, 148.9, 149.2, 150.2, 150.5, 162.5, 168.3, 173.0; FAB-MS m/e 880(M^+^); Anal calcd for C_48_H_46_ F_6_N_8_O_2_: C 65.44, H 5.26, N 12.72; found C, 65.83, H 5.39, N 12.41.

### 3.5. Cell Culture and MTT Colorimetric Assay

The human cervical cancer cell line (HeLa) was cultured with Dulbecco’s modified Eagle’s medium (DMEM, Gibco, Shanghai, China) supplemented with 10% (*v/v*) fetal bovine serum (FBS) and 1% antibiotic (100 μg/mL penicillin–100 μg/mL streptomycin, Life Technologies, Carlsbad, CA, USA) in an incubator containing 5% CO2 and 98% humidity at 37 °C, and the culture media were changed as needed. 

The cytotoxicity was investigated by MTT assay. HeLa cells were seeded in a 96-well plate at an initial density of 1 × 10^4^ cells per well in DMEM complete medium and incubated at 37 °C in 5% CO_2_ for 24 h. Then, the BPHM whose concentrations varied from 1 to 120 μM (1, 5, 10, 20, 30, 40, 50, 60 and 120 μM) were added to the medium. Each dosage was replicated in six wells. After 24 h incubation, MTT dyes (20 μL, 5 mg/mL) were added to the wells, and incubated for 4 h. The MTT solutions were removed and the formazan crystals were solubilized with 150 μL of DMSO. Then, the solution was vigorously mixed to dissolve the reacted dye. Finally, the absorbance of each well was read on a microplate reader at the wavelength of 490 nm. Cell viability (%) was calculated according to the following equation:

Cell viability (%) = A_490 (sample)_/A_490 (control)_ × 100%

where *A*_490 (sample)_ represents *A* values of the wells treated with various concentrations of BPHM, and *A*_490 (control)_ represents those of the wells treated with DMEM+10% FBS.

Each experiment was repeated three times and all of the data were analyzed by the SPSS software (SPSS Inc., Chicago, IL, USA).

### 3.6. In Vitro Cytotoxicity

The HeLa cells in photodynamic activity test were grouped into three groups: BPHM experiment groups, Mppa experiment groups and control groups. The BPHM experimental groups were treated with different concentrations of BPHM and exposed to light; the Mppa experimental groups were treated with different concentrations of Mppa and exposed to light. The dark control group remained identical to the experimental group without irradiation. About 10^4^ cells per well were seeded into 96-well plates and incubated for 24 h prepared for cytotoxicity assessment. Then, the cells of three experimental groups were rinsed with PBS, and subsequently cultured with different concentrations of BPHM and Mppa (1, 5, 10, 20, 30, 40, 50, 60 and 120 μM), total volume 200 μL per well), respectively, and then incubated for 4 h followed by exposure to visible light for 10 min (675 nm, 25 J/cm^2^), and then cultured in the dark for an additional 24 h in DMEM media as described above, 5 % CO_2_ and 98% humidity at 37 °C. Cell viability was determined by MTT assay. Each experiment was repeated three times, and all of the data were analyzed by the SPSS 19.0 software (SPSS Inc.).

### 3.7. Cellular Uptake of BPHM

*In vitro* uptake of BPHM in HeLa cancer cells was analyzed by FIM. HeLa cells were plated at a density of 10^5^ cells per well in six-multiwell plates in the DMEM growth medium. Then, they were incubated for 24 h. The medium was removed and the cells were rinsed with PBS, then incubated with 30 μM BPHM for 0.5 h, 1 h, and 3 h, respectively. Then, cells were fixed with glutaraldehyde solution (1 mL, 2.5%) for 10 min at 37 °C. The glutaraldehyde solution was removed and the cells were extensively rinsed with PBS three times again, and subsequently stained with 1 mL of 1 μg/mL DAPI nuclear probe for 10 min. Cell imaging was performed on a Leica DM IL LED Fluorescent inverted microscope (FIM) (Wetzlar, Germany).

### 3.8. Morphological Changes of HeLa Cells after PDT

Cell morphological changes were analyzed by FIM. After irradiation with light (675 nm, 25 J/cm^2^) for 10 min, the morphological changes were observed after 1 h, 3 h, 6 h, 12 h and 24 h, respectively, and the results were compared with the cells without irradiation (0 h).

### 3.9. Type I and Type II Mechanism of PDT

The test were divided into three groups: (1) BPHM-PDT groups: different concentrations of BPHM and exposed to light; (2) BPHM-PDT-SA groups: BPHM with SA (20 mmol/L) and exposed to light; (3) BPHM-PDT-DM groups, BPHM with DM (40 mmol/L) and exposed to light. Precisely, HeLa cells were seeded into 96-well plates at a cell density of 1 × 10^4^ cells per well in DMEM and incubated 24 h as described above. Then, 100 μL of different concentrations of BPHM were added to each well of 96-well plates of the BPHM-PDT groups, BPHM-PDT-SA groups and BPHM-PDT-DM groups. The final concentrations of BPHM were 1, 5, 10, 20, 30, 40, 50, 60 and 120 μM). In addition, to each well of the BPHM-PDT+SA groups were added 20 μL SA and to each well of the BPHM-PDT+DM groups were added 40 μL DM. Then, the three groups were incubated for 4 h followed by exposure to calibrated visible light (675 nm, 25 J/cm^2^) for 10 min, and then cultured in the dark for an additional 24 h in DMEM media at 37 °C under 5% CO_2_ conditions. Cell viability was determined by MTT assay. Each experiment was repeated three times, and all of the data were analyzed by the SPSS software (SPSS Inc.).

### 3.10. Statistical Analysis

Data were expressed as the mean ± SD from these independent experiments. Statistic analysis was performed using the SPSS 19.0 for Windows (SPSS Inc.). Comparisons between two groups were performed by *t*-test, and *p* < 0.05 was considered statistically significant.

## 4. Conclusions

In summary, this paper reports the preparation, spectra properties and *in vitro* photodynamic therapy of C-3^1^,13^1^ bisphenylhydrazone modified methyl pyropheophorbide-a (BPHM). *In vitro* photodynamic therapy study against HeLa cells showed BPHM had strong photodynamic effects and low dart toxicity on the cancer cells, which provided the feasibility in clinical application. BPHM could serve as an effective photosensitizer to ablate tumor cells under the illumination of the near-infrared laser (675 nm). Cell uptaking test indicated BPHM could quickly enter the cells in less than 0.5 h, suggesting that the photosensitizer could take effect more quickly in practical application, which also improved the feasibility in clinical application. We also demonstrated that the formation of reactive oxygen species in HeLa cells after treatment with PDT may be through Type I–Type II concurrent mechanism, and Type II reaction played a moderate important role. Our results indicated that BPHM has great potential applications for PDT and medical fluorescence imaging.

## Supplementary Materials

Supplementary materials can be accessed at: http://www.mdpi.com/1420-3049/21/5/558/s1.
