# Supplementary material for: A Novel Photosensitizer 31,131-phenylhydrazine -Mppa (BPHM) and Its in Vitro Photodynamic Therapy against HeLa Cells
Source: Molecules. 2016 Apr 29;21(5):558. doi: 10.3390/molecules21050558 (PMC6273471; doi:10.3390/molecules21050558)
Supplement: Supplementary file 1 [file molecules-21-00558-s001.pdf]

Supplementary Materials: A Novel Photosensitizer 3[1],13[1]-Phenylhydrazine-Mppa (BPHM) and Its *in Vitro* Photodynamic Therapy against Hela Cells

1. Statistical Analysis

Data were expressed as the mean ± SD from these independent experiments. Statistic analysis was performed using the SPSS 19.0 for Windows. Comparisons between two groups were performed by *t*-test, $p < 0.05$ considered statistically significant.

Statistical Analysis Results

Table S1. IC_{50} values obtained from the MTT assay (IC_{50} was used for T-test).

Experimental Groups	Times	IC_{50} Values Obtained from the MTT Assay
BPHM-PDT	1	8.899
	2	8.49
	3	10.246
MPPA-PDT	1	10.901
	2	13.447
	3	12.851
BPHM-PDT-SA	1	21.64
	2	19.155
	3	17.266
BPHM-PDT-DM	1	16.015
	2	14.285
	3	12.441

Table S2. *T*-test between BPHM experiment groups and MPPA experiment groups. (IC_{50} values were used for *T*-test).

Group		Levene's Test for Quality of Variances		*t*-Test for Equality of Means						
		F	Sig.	t	df	Sig.(2-Tailed)	Mean Difference	Std. Error Difference	95% Confidence Interval of the Difference: Lower	95% Confidence Interval of the Difference: Upper
Sample 1	Equal variance assumed	0.386	0.568	−3.380	4	0.028	−3.0980000	0.9165060	−5.6426286	−0.5533714
	Equal variance not assumed			−3.380	3.607	0.033	−3.0980000	0.9165060	−5.7557191	−0.4402809

$p = 0.028 < 0.05$ the difference was significant.

Table S3. *T*-test between BPHM experiment groups and BPHM-PDT-SA experiment groups. (IC_{50} values were used for *T*-test).

Group		Levene's Test for Quality of Variances		*t*-Test for Equality of Means						
		F	Sig.	t	df	Sig.(2-Tailed)	Mean Difference	Std. Error Difference	95% Confidence Interval of the Difference	
									Lower	Upper
Sample 2	Equal variance assumed	1.433	0.297	−7.386	4	0.002	−10.1420000	1.3731684	−13.9545268	−6.3294732
	Equal variance not assumed			−7.386	2.681	0.007	−10.1420000	1.3731684	−14.8217601	−5.4622399

$p = 0.002 < 0.05$ the difference was significant.

Table S4. T-test between BPHM experiment groups and BPHM-PDT-DM experiment groups. (IC_{50} values were used for *T*-test).

Group		Levene's Test for Quality of Variances		*t*-Test for Equality of Means						
		F	Sig.	t	df	Sig.(2-Tailed)	Mean Difference	Std. Error Difference	95% Confidence Interval of the Difference	
									Lower	Upper
Sample 3	Equal variance assumed	0.690	0.453	−4.340	4	0.012	−5.0353333	1.1602667	−8.2567501	−1.8139166
	Equal variance not assumed			−4.340	2.988	0.023	−5.0353333	1.1602667	−8.7361662	−1.3345004

$p = 0.012 < 0.05$ the difference was significant.

Table S5. T-test between BPHM-PDT-SA experiment groups and BPHM-PDT-DM experiment groups. (IC_{50} values were used for *T*-test).

Group		Levene's Test for Quality of Variances		*t*-Test for Equality of Means						
		F	Sig.	t	df	Sig.(2-Tailed)	Mean Difference	Std. Error Difference	95% Confidence Interval of the Difference	
									Lower	Upper
Sample 4	Equal variance assumed	0.130	0.737	3.127	4	0.035	5.1083333	1.6336092	0.5727070	9.6439597
	Equal variance not assumed			3.127	3.843	0.037	5.1083333	1.6336092	0.4987857	9.7178810

$p = 0.035 < 0.05$. the difference was significant.

2. Dynamic Light Scattering (DLS) Measurement

BPHM possessed the hydrophobic character due to the phenylhydrazine structure, hence it may agglomerate when subjected to aqueous solution. In order to investigate how BPHM enters cells (in molecular states or nanoparticles), we performed dynamic light scattering (DLS) measurement in phosphate buffer saline.

Brookhaven Instruments

Basic DLS Report

Sample		Results	
Type:	DLS	Eff. Diam. (nm):	155.07
Sample ID:	BPHM - 1	Polydispersity:	0.268
Operator ID:	Unknown Operator		
SOP ID:	20151005		
Start Date/Time:	2016/3/24 9:44:37		
Notes:	C2H5OH		

Lognormal Distribution:

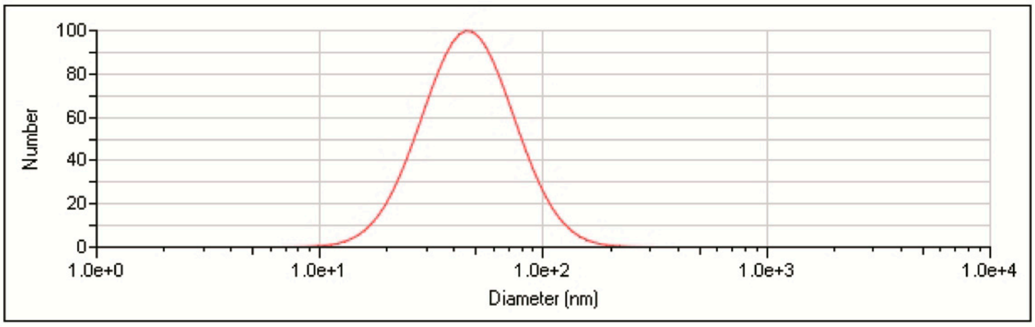

Figure S1. Dynamic light scattering (DLS) measurement in phosphate buffer saline.
